# 104 Anaylsis of positive rate among contact screening for carbapenemase-producing Enterobacterales by room and carbapenemase type

**DOI:** 10.1017/ash.2026.10522

**Published:** 2026-06-23

**Authors:** Geneva Wilson, Susheel Reddy, Muhammad Dhanani, Adovich Rivera, Deja Glover, Salva Balbale, Mackenzie Keintz, Katie Suda, Jasmine Marcelin, Charlesnika Evans

**Affiliations:** 1 Edward Hines Jr. VA Hospital; 2 Division of Infectious Diseases, Feinberg School of Medicine, Northwestern University; 3 Northwestern University; 4 Northwestern Medicine; 5 University of Nebraska Medical Center; 6 University of Pittsburgh School of Medicine; 7 Northwestern University / Department of Veterans Affairs

## Abstract

**Background:** Urinary tract infections (UTI) are common in the outpatient setting and a key focus of antimicrobial stewardship efforts. Inappropriate antibiotic prescribing persists, including low utilization of first-line agents. Limited research exists evaluating the drivers of variations in antibiotic prescribing for UTI. This analysis assessed associations between patient, clinician, and neighborhood factors and receipt of a first-line antibiotic for UTI. **Methods:** A cross-sectional analysis of adult (18 and over) urgent care visits from January 2023 to April 2025 in an integrated academic healthcare system was conducted, assessing antibiotic prescriptions for uncomplicated UTI visits. First-line antibiotics were defined as nitrofurantoin, trimethoprim-sulfamethoxazole, and fosfomycin according to IDSA clinical guidelines. Only visits associated with an antibiotic prescription were included in the analysis. Patient health status was assessed using the Charlson Comorbidity Index (CCI). Neighborhood factors were assessed using the Area Deprivation Index (ADI). Clustered logistic regression models were fit to assess factors associated with the likelihood of receiving first-line antibiotics. **Results:** A total of 13,092 UTI visits were included, of which 58% received first-line antibiotics. Patients were primarily White (73.7%), female (89.4%), and used commercial insurance (56.9%). The average age was 54.1 years, and the average CCI was 1.9. Age was associated with a lower likelihood of receiving first-line therapy; each additional year of age (<18) was associated with a 1% decrease in odds (Adjusted Odds Ratio (aOR) = 0.99, 95% CI = 0.98, 0.99). Similarly, each one-point increase in CCI decreased the likelihood of receiving first-line antibiotics by 4% (aOR = 0.96, 95% CI = 0.94, 0.97). Compared with men, women were less likely to receive first-line antibiotics (aOR=0.55, 95% CI=0.48, 0.62). Treatment by a physician assistant (PA) (aOR=0.91, 95%CI=0.83, 0.98) or a registered nurse (APRN) (aOR=0.66, 95%CI=0.60, 0.73) was associated with a reduced likelihood of first-line medication. Finally, being on Medicaid (aOR=0.82, 95% CI=0.71, 0.94) or Medicare (aOR=0.82, 95% CI=0.73, 0.91) was associated with a decreased likelihood of receiving first-line antibiotics. No statistically significant association was found between living in a more disadvantaged neighborhood, as measured by the ADI, and receiving first-line antibiotics. Discussion: Being older, female, in poorer health, treated by a PA or APRN, or having Medicare/Medicaid were associated with a lower likelihood of receiving first-line antibiotics for UTI. These findings provide critical information to understand variations in antibiotic prescribing for UTI. Further work is needed to identify the drivers of these differences to improve equity in stewardship.

